# Validation of a complex needs indicator for veterans in the UK using a composite indicators’ method

**DOI:** 10.1016/j.puhip.2024.100464

**Published:** 2024-01-05

**Authors:** Anastasia Fadeeva, Marco Tomietto, Ajay Tiwari, Emily Mann, Giuseppe Serra, Matthew D. Kiernan

**Affiliations:** a Faculty of Health and Life Sciences, Northumbria University, Newcastle upon Tyne, United Kingdom; b Violence and Society Centre, City, University of London, London, United Kingdom; c Centrum Wiskunde & Informatica, Amsterdam, Netherlands; d Department of Medicine (DIME), University of Udine, Udine, Italy

**Keywords:** Veterans, Complex needs, Beneficiaries, Composite indicators, Social care

## Abstract

**Objective:**

To construct an indicator for assessing the complexity of UK veterans’ needs.

**Study design:**

Cross-sectional, secondary analysis.

**Methods:**

The study applied principal component (PCA) analysis as the method to determine the weights of different needs based on their interactions with each other, the effectiveness of the model was evaluated using bootstrapping. The dataset on UK veterans’ support provided by the “Soldiers, Sailors, Airmen and Families Associations” (SSAFA) (N = 35,208) was considered. The grant applications for different categories of support were used as indicators of different needs. The dimensions of breadth (number of different needs) and depth (number of grant applications to address the need) were incorporated in the assessment of complexity.

**Results:**

The complex needs indicator for the current sample was validated. The majority of cases had a complexity score of 1 or less.

**Conclusions:**

The research suggested and tested an assessment method for the complexity of veterans’ needs, that may be positively associated with higher risk of adverse health outcomes. This indicator can be used by decision-makers for risk stratification of the veteran population, thus supporting the allocation of resources in a more effective way.

## 1 Introduction

In 2017 there were 2.4 million UK Armed Forces veterans in the United Kingdom (UK), and 49% of veterans are aged 75 years and over [1]. The most recent 2021 Census [2] identified that there were 1.85 million veterans in England and Wales (the census did not cover Scotland and Northern Ireland) and 7% of all households had one or more person that had served in the UK Armed Forces. Consequently, it is projected that by 2028, the total number of veterans will fall to 1.6 million with the demographic profile of working (44%) and non-working or retired (56%) veterans becoming more balanced as a result of a decline in the non-working veteran (retired) group. The population of veterans over 75 years is projected to decline in number to 37% [3]. Despite the contracting population, there appears to be a growing demand for support for complex needs within the veteran charity sector.

Veterans are a particularly vulnerable population in terms of health. During active duty they are exposed to several risk factors, the effect of which often becomes evident only when they leave service. Some of these risks are directly related to service, such as physical risk (e.g., noise), chemical risk (e.g. heavy metals) and biological risk (e.g. endemic infectious diseases), as well as direct injury or death during combat [3]. In addition, studies have shown how unhealthy behaviours like heavy drinking, smoking and illicit drug consumption have a high prevalence in active military personnel [4].

On top of these risk factors, the stress of being in service can lead to psychological sequelae such as Post-Traumatic Stress Disorder (PTSD), depression and anxiety [5]. Mental health issues are less likely to be reported by veterans than the rest of the population and in turn are associated with other risk factors for cardiovascular disease such as hypertension, dyslipidaemia, obesity, and diabetes [6]. It is suggested that one component of this recent scenario might be the result of improvements in the survival rates following major trauma in recent UK combat operations in Iraq and Afghanistan along with advances in health care [4]. Approximately 14,000 service men and women leave the armed forces each year and for some the transition to civilian life is affected by economic struggle, unemployment and food insecurity [[8], [9], [10]]. These factors are widely known as social determinants of health [11]. Furthermore, these health and social issues interact with each other, resulting in a growing number of veterans experiencing complex needs where increased complexity may lead to poor health outcomes. When multiple risk factors are present in an individual, the combined effect may differ from the simple sum of each individual effect (known as “effect modification”) [8]. Therefore, the veteran population carries significant morbidity and complexity, while the effect on mortality is still uncertain.

## 2 Background

Rankin and Regan [5] defined complex needs as having more than one issue and introduced the notions of the ‘breadth of need’ (existence of multiple needs that are interconnected) and ‘depth of need’ (severity and intensity of need). More recently, the All Party Parliamentary Group (APPG) on Complex Needs and Dual Diagnosis has defined someone as having complex needs as having a co-morbidity of two or more of the needs shown in Table 1 and those needs will usually be severe and longstanding [6].

**Table 1:** Defining complex needs.

Mental health issues	Substance misuse issues	A diagnosis of mental health and substance misuse issues	A physical health condition	A learning disability	A history of offending behaviour	A physical disability
Employment problems	Homelessness or housing issues	Family or relationship difficulties	Domestic violence	Social isolation	Poverty	Trauma (physical, psychological or social

Despite the vast impact of complex needs on individuals, society and health and social care, there is paucity of research on populations in the UK at risk of complex needs and no research has attempted to estimate the trends on complex needs among veterans. Understanding complex needs presents a number of challenges due to the diversity of their nature, difficulties of accessing, lack of information, and because individuals with complex needs often are the hardest to reach [7].

In response to the aforementioned challenges, the assessment solution should derive from the understanding of what complex needs are. As defined by Rankin and Regan [12], the breadth of the need is presented by the range or number of needs an individual has. The depth of need is potentially harder to measure and compare, as its assessment is due to subjective individual and service measurements. However, as an individual interacts with support services, an indicator of severity could include the amount of time required to manage a case, the number of requests for support, and, or, the number of interventions required to address the needs over time. Additionally, Rankin and Regan emphasised the importance of assessing the interaction between needs to understand their complexity. This is aligned with complexity theory that recognises connectivity and interdependence between factors in a system. The principles of complexity and the importance of considering multimorbidity, along with interacting sociocultural influences, have been more widely acknowledged in contemporary health and social care [8].

An increasingly popular way to measure complex phenomena is the use of composite indicators. A composite indicator is a synthetic index of individual indicators that are complied on the basis of one underlying model or multi-dimensional system that is being measured [9]. Composite indicators account for weighting of the sub indicators and interaction between them and summarise complex information in a single digit. This makes it easier to understand and compare the cases. Given the interconnection between complex needs and differences in their “importance” is critical in determining the potential of those needs to deepen or cause other issues that would have an impact on an individual’s life situation. The methods used in the development of composite indicators are therefore important in estimating the complexity of veterans’ needs.

With regards to the input data that can be used to understand veterans’ need and assess their complexity, a more reliable and comprehensive approach is to use the information from organisations that address various needs of the veterans and collect data on multiple cases. Military charities provide a broad range of services and support in different domains (e.g., financial, health) to the veteran community in the UK, the information of which can be used as a proxy measure for measuring complex needs.

A protocol for developing a complex needs indicator for veterans has been previously published to address the methodological aspects of complexity and to present a method to identify the components and the weight of complexity for each component [10]. This study aims to test and further develop the method for constructing a complex need indicator for UK veterans, by considering the requests for support received by the charities in the sector.

## 3 Methods

### 3.1 Study design

Cross-sectional, secondary analysis.

### 3.2 Study population

All participants included in this study were UK veterans (N = 35,208; *M*_age_ = 57.00, *SD* = 19.17, *Female* = 4660).

### 3.3 Data collection

The “Soldiers, Sailors, Airmen’s and Families Associations” (SSAFA) data related to benefit cases and number of beneficiaries applied for grant support have been collected through a yearly average count between 2014 and 2019. SSAFA manages means tested financial and social support for over 128 military charities and organisations and collects beneficiary data on their behalf in a case management system. This makes the organisation being the most comprehensive data source representing the veteran population in the UK. Since the charity performs a comprehensive assessment of the needs (by considering health, social, and financial aspects) before awarding services, it can be presumed that all the needs of a subject are recorded in the database.

### 3.4 Variables

To explore the complexity of cases, we were guided by the definitions provided by the APPG on Complex Needs and Dual Diagnosis, and Rankin and Regan [12]. The “breadth” of needs was represented by the number of various needs each beneficiary had. As a proxy measure of veterans’ needs, we used the data on their applications for different types of support (e.g., mobility assistance, home fixtures and fittings, support with food, rent or utility bills etc.) from SSAFA. The grants were categorised in to needs in accordance with the adapted dimensions suggested by the APPG (Table 2). The number of applications for different types of support by beneficiaries over time was used to represent the “depth” of need.

**Table 2:** Grants categorisation by needs.

Grants	Need category
**Care Charges (care at home)** **OT Charges** **Local Authority Social Services** **Carephone** **Maintenance Grant (all other)** **Maintenance Grant (care home)** **Maintenance Grant (all other)** **Maintenance Grant (care home)** **Respite Breaks (W + B)** **Care Homes** **Handy Van & Carephone**	Care
**Children needs**	Children needs
**Debt (bankruptcy fees)** **Debt (non-priority)** **Debt (priority)** **Housing (repairs and maintenance) - Grant** **Housing (repairs and maintenance) - Loan** **Housing (gardening)** **Immigration or Visa Fees** **Travel Costs (clients)** **Family & Adventure Breaks** **Insurance** **Funeral Costs** **Deposit Guarantee** **Benefits & Tax Credits** **Benefits & Money Advice** **Deposit Guarantee**	Financial/Debt
**Essential Clothing**	Essential Clothing
**Essential Food and Groceries** **Foodbank**	Essential Food
**General Needs (discretionary)**	General Needs
**Household Goods (brown)** **Household Goods (white)** **Essential Household Appliances**	Household
**Housing (damages and arrears)** **Housing (deposits and charges)** **Housing (removal expenses)** **Housing (rent)** **House purchases** **Rent Review (RAFBF only)** **Local Authority Housing**	Housing
**Medical (dental charges)** **Medical (optician charges)** **Medical (other)** **National Health Service**	Medical
**Mobility Fixtures** **Mobility Home Adaptation** **Mobility/EPV (storage and access)** **Mobility/EPV (vehicles)** **Motability Scheme Deposit** **Stairlifts (purchase)** **Stairlifts (rental charges)** **Riser/Recliner & Electric Beds**	Mobility
**Counselling** **Combat Stress**	Mental Health
**Legal Fees** **Citizens Advice**	Legal
**Training Costs - Fees** **Training Costs - Materials** **Traning Costs - Materials** **Job Centre Plus**	Education/Employment

### 3.5 Estimating needs weights

As needs tend to interact and exacerbate each other [5], some may have a greater “importance” in terms of their likelihood to affect other needs, and subsequently lead to a complex case or presentation. Therefore, accounting for this inter-relationship between needs is an important step in measuring complexity, and a methodology to estimate weights which derive from their correlations with each other was applied. One way of estimating weights based on their correlations with each other is to apply principal component analysis (PCA). PCA has been used as a weighting method in the development of composite indicators [9]. PCA calculates the loadings of variables that contribute to the multi-dimensional phenomena by capturing the multiplicity of related variables and by maximizing the proportion of the variance in the original variables [9].

### 3.6 Data analysis

Principal component analysis was applied for estimating the weights of the needs [11]. Each need category incorporated various support that SSAFA beneficiaries applied for (Table 2). The need categories and classification of support were based on the nature of the problems for which SSAFA granted assistance and were agreed through discussion with the charity. The grant applications that belonged to the same need categories were aggregated together. The needs were then aggregated by beneficiary service number and date of birth to account for the complexity of needs within individuals and were then used as variables in PCA.

Mahalanobis distances and their p-values of chi-square statistics with 13 degrees of freedom were measured to identify multivariate outliers and to check the multivariate normality [12]. Mardia's kurtosis index was found to be 8743.44, which was above the threshold value of 195 [13], stating the non-normality of the distribution. After removing outliers (n = 2154) the Mardia's kurtosis index was 34.57. However, by deleting multivariate outliers in order to achieve multivariate normality, two categories of needs were deleted (mental health and legal support). In order to retain as much information as possible to properly represent the complexity of needs, the outliers were kept, as cases with the extreme scores could still represent individuals with complex needs [13].

### 3.7 Determining weights of complex needs

The selected variables were normalised using Min-Max transformation (see Equation (1)) before proceeding with the next steps of the analysis.

Equation 1 $$TX_{i}=\frac{X_{i}-X_{iMin}}{X_{iMax}-X_{iMin}}$$

where *TX*_*i*_ is the transformed value of the original variable *X*_*i*_, *X*_*iMax*_ and *X*_*iMin*_ are the maximum and minimum values of the original variable *X*_*i*_ respectively.

Before applying PCA, a Multicollinearity check was performed using the Kaiser-Meyer-Olkin (KMO), which is a measure of sampling adequacy and the Bartlett’s test of sphericity. The calculated KMO value of 0.59 was above the accepted cut-off point of 0.50 and the Barlett’s Sphere Test (*λ*^*2*^ = 12410.377; *df* = 78; *p* < .0001) indicate the suitability of the sample size for performing the PCA. PCA was used as the extraction method and the components were rotated with the varimax technique, which minimised the number of indicators with high loadings on each component. To determine the total number of principal components to be extracted for the dataset in PCA, the parallel analysis was implemented as a more accurate alternative [14] to the Kaiser [15] rule. After applying PCA, the factor loadings of all the retained factors were considered, which enabled the preservation of the largest proportion of the variation in the original dataset. The factors’ loadings were then squared and scaled to unity sum to calculate the final weights [16].

The calculated weights and the number of grant applications over the study period were then used as multipliers for corresponding needs, which were aggregated to calculate a total complex need score (Equation (2)).

Equation 2 $$CNS_{i}=\sum_{v=1}^{v}W_{{PCA}_{n}}S{VI}_{in}$$

where *CNS*_*ί*_ is the complex needs score for an individual *‘ί’, W*_*PCAn*_ is the weight of the *CNS*_*ί*_ sub indicator or need category obtained from the PCA, *SVI*_*ίν*_ is a number of grant applications for each need category *‘n’* for an individual *‘ί’*. The analyses were performed using MS Excel and R (version 4.1.0).

### 3.8 Ethical considerations

Data have been stored according to the University of Northumbria at Newcastle policy and practice, which complies with Data Protection Act 2018 and incorporates General Data Protection Regulations (GDPR) [17]. Ethical approval has been provided by Northumbria University (reference number 0768).

## 4 Results

Following the application of the aforementioned methodological steps, the complex needs indicator for the sample was constructed. The majority of beneficiaries had a complex score of 1 or less.

The parallel analysis suggested that five components should be retained for correlation matrix PCA loadings. The components were then extracted using varimax rotation. Table 3 presents factor loadings of the needs.

**Table 3:** Factor loadings of complex needs based on principal components.

	Factor loading					Squared factor loading (scaled to unity sum)				
	1	2	3	4	5	1	2	3	4	5
**Essential Food**	**.66**	−.09	.15	−.08	−.12	**.29**	.01	.02	.01	.01
**Essential Clothing**	**.61**	−.05	−.16	.06	.20	**.25**	.00	.02	.00	.04
**General Needs**	**.61**	.04	.11	−.05	−.31	**.25**	.00	.01	.00	.09
**Children Needs**	**.47**	.03	.02	.18	.29	**.14**	.00	.00	.03	.08
**Mobility**	−.15	**.70**	−.03	.00	.03	.02	**.36**	.00	.00	.00
**Care**	.12	**.69**	−.04	−.01	.04	.01	**.35**	.00	.00	.00
**Financial/Debt**	.09	.05	**0.72**	.00	−.05	.01	.00	**.45**	.00	.00
**House Rent**	.10	−.38	**.49**	−.12	.10	.01	.11	**.21**	.01	.01
**Legal**	−.03	−.02	**.45**	.09	.03	.00	.00	**.17**	.01	.00
**Mental Health**	−.04	−.08	.04	**.76**	.08	.00	.01	.00	**.53**	.01
**Medical**	.07	.09	.04	**.66**	−.14	.00	.01	.00	**.40**	.02
**Employment/Education**	.10	−.27	−.26	.10	**−.70**	.01	.05	.06	.01	**.45**
**Household**	.13	−.40	−.26	−.05	**.56**	.01	.12	.06	.00	**.29**
Explained Variance	1.49	1.37	1.16	1.09	1.08					
Explained Total, %	24	22	19	18	17					

*Note.* Estimated weights for the needs are in bold.

**Fig. 1:**
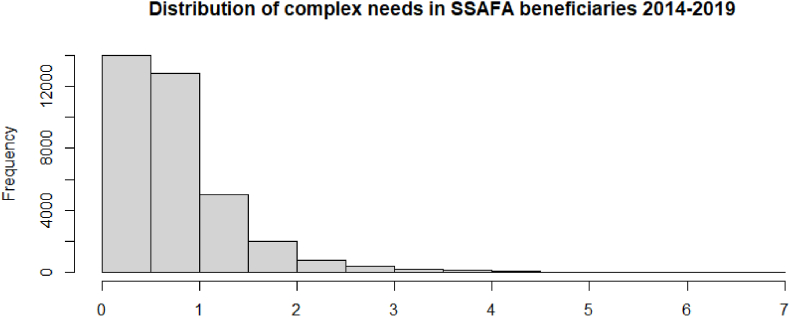
Fig. 1. Distribution of complex needs in SSAFA beneficiaries 2014-2019.

The final weights were then calculated using the absolute values of the squared loadings of the variables on each dimension (Table 3). Table 3 shows that the first component accounts for 24% of total variance, has high positive coefficients (loadings) with essential food (0.66), essential clothing (0.61), general needs (0.61), and children needs (0.47). The second component accounts for 22% of total variance, and it includes mobility (0.70) and care (0.69) needs. The third component accounts for 19% of the total variance and has high loadings with financial hardship and debt (0.72), house rent (0.49), and legal support (0.45). The fourth component accounts for 18% of total variance and is formed by mental health (0.76) and medical needs (0.66). Finally, the fifth component accounts for 17% of the total variance and is dominated by household (0.56) and employment and educational needs (−0.70). These weights were then put in Equation (2) to calculate the complexity need score for each individual. The distribution of scores for the SSAFA beneficiaries in 2014–2019 is presented in Fig. 1. The higher score indicates a greater complexity of needs. The results suggest that the majority of beneficiaries had lower complexity of needs, with more than 25,000 individuals having a complex score of 1 or lower. Given the estimated weights of needs (i.e., 0.47–0.76), this implies that most beneficiaries applied for support for one or two needs during the study period. However, the top 1% of the study sample had a complex need score of 3.04 or higher with a maximum score of 6.83, which suggest there was still a proportion of beneficiaries with complex needs.

To check the robustness, the uncertainty of the model was quantified by calculating the confidence intervals for the aggregated complex need scores. The bootstrapping method was used to calculate the confidence intervals for the CNSs Level BCa 95% (Table 4) [18]. The estimated bootstrap confidence interval (95%CI 0.78–0.78) captures the mean of 0.78, estimated for the current sample, which suggest the applied statistical method is robust.

**Table 4:** Bootstrap statistics.

Level	BCa*
95%	(.78–.78)

Note. * = *p* < .05, ** = *p* < .01, *** = *p* < .001; significant changes in bold.

## 5 Discussion

The present study tested a method used for constructing composite indicators to estimate the complexity of UK veterans’ needs. Studies [4,19,20] and anecdotal concerns within the military charities sector have highlighted that veterans seeking help in recent years are presenting with more complex needs. However, to date this assertion has neither been assessed or validated. This study involved a sample from one of the largest UK military charities, that manages cases for over 128 organisations, and implemented a complex needs indicator by including both medical and non-medical variables.

The weights for thirteen different needs of UK veterans in the domains of physical and mental health, education and employment, financial hardship, food poverty, and living environment were estimated using PCA. This approach implemented the previous protocol [10] by considering the OECD guidance on composite indicators and by adopting a different data analysis approach to further refine the indicator. The present study partially deviated from the original protocol by applying PCA as the main estimation method. PCA is one of the most commonly used, and preferred approach, in the development of composite indicators, due to its simplicity and its ability to allow for the construction of weights representing the information content of individual sub indicators [9,21]. Importantly, the estimates in PCA derive from correlations between the variables, which was valuable in accounting for the interactions between different needs. Mental health had the highest weight based on the analysis. This might suggest that people who applied for mental health support were most likely to apply for other types of support as well. Mental health problems among veterans tend to be related to unemployment, economic challenges, homelessness, and other adverse societal effects [22]. Other variables with high weights were employment and education need and financial need. This study has highlighted the most relevant components of complexity. These findings have the potential to effectively address assumptions around health and social care provision planning for this population and inform wider policy with regards to veteran’s needs. Furthermore, this study provides useful insights to tailor the support offered to veterans according to their complexity level. By understanding and measuring complexity, it is possible to meet more accurately the veterans’ needs and provide access to more targeted services and support in specific areas.

### 5.1 Limitations

This study did not measure the veterans’ needs directly but considered grant applications and service usage by veterans for various types of support as an indicator of need. It is important to note that the demand for the support might not always directly indicate the actual need, and that availability of alternative forms of support in certain areas may have influenced the propensity of the veteran to ask for charity support. However, all support delivered by SSAFA is means tested and only granted following a comprehensive assessment of need. The study also relied on the data collected by volunteer workers, and the accuracy of the collected information was not possible to verify. Furthermore, whilst this study included non-medical needs in the evaluation, the data on potential predictors of complex needs such as health behaviours (i.e., alcohol consumption) were not available. As follow up of veterans was not included in the analysis, the value of the indicator is not to be interpreted as the risk of specific health outcomes, but a measure of complexity itself. This study specifically focused on testing the complex need indicator, however the dataset retrospectively covered a range of 5 years and there is the potential to track the evolution of the complexity of needs over time.

### 5.2 Recommendations for the future research

The future assessments of veterans’ needs should consider using direct measurements of needs and predictors such as questionnaires on health status, food insecurity, and substance abuse. While this study highlighted the weight of each component on the overall complexity of needs, it would be important to further test and validate the complex needs indicator by considering its capacity to predict health and social outcomes. For this purpose, a longitudinal study design would be the most appropriate. While the data source of this study is currently the most representative of the veteran population in the UK, it is not exhaustive of the entire population. For this reason, it is also recommended that to expand the study population, beneficiaries from other military charities and different services should be included. Ideally, this would be applied to an aggregated dataset of all the military charities data, to determine the complexity of need when considering how an individual accesses multiple services and organisations. This indicator would then be further refined by merging these different data flows from different charities and institutions (e.g., community health services and the healthcare system) in order to directly measure the above-mentioned components of complexity. It still remains extremely difficult to identify UK veterans and explore their needs. This study has demonstrated a method for exploring trends over time, in different geographic locations using data that is readily available within this population. Beyond the specific data sources available in the UK, this study has also provided a methodology that can potentially be adopted in other countries to represent the complexity of needs in the veteran population and, in this way, provide international insights on this area. Moreover, this study has provided a useful indicator for both retrospective and prospective mapping of changes in the complexity of needs in this population and developing predictive models over time.

## 6 Conclusions

The study described an assessment method for veterans’ needs, which can be potentially further employed to evaluate the distribution of complex needs among veterans, measure changes in complexity over time, and identify geographic regions with more complex cases. Once adopted, the indicator could support decision-makers to effectively address resources to areas at higher risk and complexity. An early intervention could mean less adverse events, lower cost, and ultimately an overall improvement of veterans’ health.

## Authors’ statement

All authors have made key contributions to the conception, design, drafting and review of the manuscript. Specifically, the authors [blinded, please see the cover letter] contributed to the conception and design of this study. The authors [blinded, please see the cover letter] contributed to the protocol. The authors [blinded, please see the cover letter] have drafted and reviewed the current manuscript and agree with its submission.

## Ethical approval

Ethical approval has been provided by Northumbria University (reference number 0768).

## Funding

This project was funded by the Armed Forces Covenant Fund Trust, grant number: CF-SG001.

## Declaration of competing interest

The authors declare that they have no known competing financial interests or personal relationships that could have appeared to influence the work reported in this paper.
